# Fine Particle, Ozone Exposure, and Asthma/Wheezing: Effect Modification by Glutathione S-transferase P1 Polymorphisms

**DOI:** 10.1371/journal.pone.0052715

**Published:** 2013-01-24

**Authors:** Bing-Fang Hwang, Li-Hao Young, Ching-Hui Tsai, Kuan-Yen Tung, Pei-Chuan Wang, Ming-Wei Su, Yungling Leo Lee

**Affiliations:** 1 Department of Occupational Safety and Heath and Graduate Program, College of Public Health, China Medical University, Taichung, Taiwan; 2 Institute of Epidemiology and Preventive Medicine, College of Pubic Health, National Taiwan University, Taipei, Taiwan; Vanderbilt University Medical Center, United States of America

## Abstract

**Background:**

There are limited studies on the role of interaction between exposure to ambient air pollution and glutathione-S-transferase (GST) P1 on the risk of asthma/wheezing among children, which provided suggestive, but inconclusive results.

**Methods:**

To assess the joint effect of air pollutants and GSTP1 on asthma/wheezing, we conducted a nationwide cross-sectional study of 3,825 children in Taiwan Children Health Study. The studied determinants were three GSTP1 Ile105Val (rs 1695) genotypes (Ile-Ile; Ile-Val and Val-Val) and expoure to ambient air pollutants. We used routine air-pollution monitoring data for ozone (O_3_) and particles with an aerodynamic diameter of 2.5 µm or less (PM_2.5_). The effect estimates were presented as odds ratios (ORs) per interquartile changes for PM_2.5_ and O_3_.

**Findings:**

In a two-stage hierarchical model adjusting for confounding, the risk of asthma was negatively associated with PM_2.5_ (adjusted odds ratio (OR) 0.60; 95% confidence interval (CI) 0.45, 0.82) and O_3_ (OR 0.74; 95% CI 0.60, 0.90) among Ile105 homozygotes, but positively associated with PM_2.5_ (OR 1.52; 95% CI 1.01, 2.27) and O_3_ (OR 1.19; 95% CI 0.91, 1.57) among those with at least one val105 allele (interaction p value = 0.001 and 0.03, respectively). A similar tendency of effect modification between PM_2.5_ and O_3_ and GSTP1 on wheezing was found.

**Conclusion:**

Children who carried Ile105 variant allele and exposed to PM_2.5_ and O_3_ may be less likely to occurrence of asthma/wheezing.

## Introduction

Both genetic and environmental factors play important roles in the aetiology of asthma and wheezing. There is probably also genetic susceptibility to the effects of air pollution [1]. Identification of indicators of genetic susceptibility to environmental exposures could be useful from preventive point of view [2]. Recent studies of genes-environment interaction or effect modification for asthma mainly focused on members of the glutathione-S-transferase (GST) superfamily because several members, particular in glutathione-S-transferase p1 (GSTP1), glutathione-S-transferase m1 (GSTM1) and glutathione-S-transferase t1 (GSTT1), are expressed in the respiratory tract and function in processes implicated in asthma pathogenesis, including oxidant defenses, xenobiotic metabolism, and detoxification of hydroperoxides. DNA sequence variants in the GSTP1 Ile105Val locus, GSTM1 null, and GSTT1 null may contribute to susceptibility to oxidative stress and airway inflammation, which are key processes in asthma pathogenesis [3].

In our systematic Medline search, only four studies concerning effect modification betwen exposure to ambient air pollution and GSTP1 on childhood asthma/wheezing provided inconclusive results [4]–[7]. Melen and colleagues conducted a birth cohort study of 4,089 children in Stockholm, where they reported reduced risks of asthma and persistent wheezing among children with Ile105 homozygotes for NOx exposure during the first two years of life compared with at least one Val105 allele, but the interaction effect was not significant (p-value for interaction p>0.05) [4]. Islam and colleagues conducted a cohort study of 1610 school children in Southern Caifornia indicated negative but not significant association between the risk of asthma and high ozone communities (range from 46.5 to 64.9 ppb) among at least Val105 homozygotes with an adjusted hazard ratio of 0.98 (95% CI 0.4–2.3) [5]. Schroer and colleagues conducted a birth cohort study in Cincinnati, Ohio found children carrying the Ile105 allele may confer protection from wheezing, but exposure to high level of diesel exhaust particle (DEP≥0.5 ug/m3) converging on a similar pathway may overwhelm the genetic effect [6].

Our previous study suggested an interaction between GSTP1 and outdoor air pollution on childhood asthma [7]. This study did not assess the role of air pollutants such as particles with an aerodynamic diameter of 2.5 µm or less (PM_2.5_), and ozone (O_3_). Taiwan Children Health Study (TCHS) offers an opportunity to investigate the joint effects of ambient air pollution exposure and GSTP1 on childhood asthma and wheezing. At the cohort entry, we collected information of allergic symptoms during the past 12 months and also on those important potential determinants of allergic disease in children. In the present study, we elaborated the effect modification relations between exposure to air pollution and GSTP1 on the risk of asthma and wheezing in school children, focusing on PM_2.5_, and ozone (O_3_). Further we applied a two-stage hierarchical model to adjust for confounding and to elaborate effect modification on individual-level and to assess the effects of air pollution between communities [8]–[9].

## Methods

### Data collection and study population

Taiwan Children Health Study (TCHS) was based on a multi-purpose nationwide design that focused on outdoor air pollutants as primary interest. Communities of Taiwan were selected with the aim of maximizing the variability and minimizing the correlations in criteria outdoor pollutants based on historic routine air monitoring data. A total of 4,102 seventh grade children were recruited from public schools in 14 communities covering diverse parts of Taiwan, which was representative of Taiwanese middle-school children in 2007. The study detail has been described previously [10]. We finally approached 3,816 seventh-grade school-aged children who completed the standard questionnaire and sufficient genetic information (response rate 93.0%).

The study protocol was approved by the Institutional Review Board (National Taiwan University Hospital Research Ethics Committee; number: 200902042R), and complied with the principles outlined in the Helsinki Declaration [11]. We have informed all the participating children/parents and obtained their written consent forms in this study.

### Health outcomes

The outcomes of interest were the onsets of asthma and wheezing before the ambient air pollution in recent three years. The definition of asthma subjects was determined by a positive response to the question “Has a physician ever diagnosed your child as having asthma?” Five questions related to current asthmatic symptoms were also asked:

1. In the past 12 months, has your child had dyspnoea with wheezing in the chest? (wheezing)
2. In the past 12 months, has your child's sleep been disturbed because of wheezing? (night wheezing)
3. In the past 12 months, has wheezing ever been severe enough to limit your child's speech to only one or two words at a time between breaths? (dyspnoea at rest)
4. In the past 12 months, has your child's sounded wheezy during or after exercise? (exercise wheeze)
5. In the past 12 months, has your child had a dry cough at night, apart from a cough associated with a cold or chest infection? (night cough)

The criteria of non-asthmatic subjects was (i) no physician diagnosed asthma or dyspnoea with wheezing in the past; (ii) no positive response to any of the five questions concerning current asthmatic symptoms. The definition of wheezing was determined by a positive response to the question “Has your child ever had wheezing or whistling in the chest at any time in the post when he/she did not have a cold or flu?” Non-wheezing subjects were defined on the basis of these reporting not ever having dyspnoea with wheezing, no nocturnal dyspnoea associated with wheezing and without physician diagnosed asthma.

### Genetic polymorphisms analysis

Cotton swabs containing oral mucosa were collected and were stored at −80°C before analysis. Genomic DNA was isolated using phenol/chloroform extraction method. The polymorphism of GSTP1Ile105Val, GSTM1 (null/presnt) and GSTT1 (null/present) were detected by real-time polymerase chain reaction using the TaqMan Allelic Discrimination assay on an ABI PRISMTM 7900 Sequence Detector (Applied Biosystems, Foster City, CA) [12]. The sequences of primers and MGB probes used are listed in Table S1. Each subject's clinical status remained anonymous to laboratory staff and genotype assignments were based on two consistent experimental results. About 15% of the randomly selected samples were sequenced, and all of them were consistent with the initial genotyping results.

### Exposure assessment

Complete monitoring data for the air pollutants including sulphur dioxide (SO_2_), nitrogen dioxides (NO_2_), ozone (O_3_), carbon monoxide (CO), and particles with an aerodynamic diameter of 2.5 µm or less (PM_2.5_) are available for 14 EPA monitoring stations in TCHS communities. Concentrations of each pollutant are measured continuously and reported hourly—CO by non-dispersive infrared absorption, NO_2_ by chemiluminescence, O_3_ by ultraviolet absorption, SO_2_ by ultraviolet fluorescence, and PM_2.5_ by beta-gauge. Exposure parameters in the present study were 3-year average (2005–2007) and the yearly deviations from the 3-year average concentrations in each municipality, calculated from the 24-hour NO_2_, CO, SO_2_, PM_2.5_, and 10:00 AM to 6:00 PM 8-hour O_3_.

### Covariates

Information on potential confounders was obtained from the parental-administered questionnaire. The covariates in the present analyses included age, gender, parental education, family annul income, duration of breast feeding, maternal smoking history during pregnancy, environmental tobacco smoke (ETS), cockroaches note, carpet used, home dampness and mould, and parental atopy. Parental atopy was a measure of genetic predisposition to asthma and it was defined as the father or mother of the index child ever having been diagnosed as having asthma, allergic rhinitis, or atopic eczema.

### Statistical methods

The Hardy-Weinberg equilibrium was calculated for the all polymorphisms in our study population using Pearson χ^2^ statistics. We estimated adjusted odds ratios in a two-stage hierarchical model using logistic and ecologic model analyses. The models assume two sources of variation: the variation among subjects in the first stage, part of which could be explained by the individual confounders, and the variation of air pollution between communities in the second stage, part of which could be explained by variables measured at municipal level. In the analyses we assumed that 1) the outcome variable follows Bernoulli distribution; 2) intercept terms are random at the municipal level; 3) all the explanatory variables are fixed effects. A logistic regression model was fitted in the first stage for the risk of bronchitic symptoms as a function of site-specific intercepts, j, where αj = 1, …, 14, and personal covariates. The adjusted site-specific intercepts and prevalence rates are related by Pj = e^αj^/(1+e^αj^). In the second stage, these intercept terms representing the logit of the site-specific prevalence rates (Pj; j = 1, …, 14), adjusted for personal covariates, were regressed on each site-specific ambient pollutant level by using a linear “ecologic” regression, i.e., logit αj = α+Uj+βZj, where Uj denotes the random departure from the general prevalence αj on the logit scale for site j; Zj denotes the ambient pollution level for site j. Thus, β can be interpreted as the log odds ratio (per interquartile changes) for each pollutant, adjusted for personal characteristics. The results from the models are presented as odds ratios (ORs), along with their 95% confidence intervals (CIs). The goodness of fit was assessed with likelihood ratio tests (LR) to determine whether a variable contributed significantly to the model. First, we fitted a full model with a complete set of covariates. To elaborate sources of confounding, we fitted models with different combinations of covariates and compared the effect from models with and without the covariate of interest. If the adjusted odds ratio differed from the crude odds ratio by more than 10%, that covariate was included in the final model. The two-stage hierarchical model was used not only to derive more precise estimates of site-specific parameters and site-level effects, but also to adjust for multiple comparisons [13].

We considered the effect of multiple pollutants on the risk of asthma/wheezing. We first fitted one-pollutant models, and then considered two-pollutant models by fitting one (NO_2_ or CO) and the other (SO_2_ or PM_2.5_) pollutant. Then, we fitted two-pollutant models with O_3_ or PM_2.5_ and another pollutant. It was not appropriate fit two-pollutant models with O_3_ and PM_2.5_ because of high collinearity (correlation coefficient r = 0.73). The two-pollutant models provide estimates of the independent effects of PM_2.5_, and O_3_ on the asthma/wheezing controlling for the second pollutant in the model. The effect of each pollutant on the risk of asthma/wheezing was presented as odds ratios (ORs) per interquartile changes for O_3_, and PM_2.5_.

We considered the joint effect between air pollutants and GSTP1, GSTM1, or GSTT1 on the risk of asthma and wheezing introducing interaction terms in the model. The community specific average air plloutnats levels were fitted as continuous variables along with the appropriate interaction terms for GSTP1, GSTM1, or GSTT1 variant and air pollution levels. All tests assumed a two-sided alternative hypothesis and a 0.05 significance level.

## Results

### Characteristics of study population

Hardy-Weinberg equilibrium tests for GSTP1 showed non-significance (p>0.05) in both asthmatic and non-asthmatic groups as well as wheezing and non-wheezing groups. The characteristics of the study population according to the baseline covariates are shown in Table 1. The higher proportion of asthmatic subjects was male and had maternal smoking during pregnancy, any home dampness and mould, gestational age (<37 weeks) and parental atopy. Besides above covariates, the larger proportion of wheezing subjects was also related to the presences of cockroaches and duration of breastfeeding. We adjusted for these factors in the multivariate analysis.

**Table 1:** Distribution of demographic and other characteristic of study subjects.

Characteristic	Asthmatic subjects (n = 295)	Non-asthmatic subjects (n = 3517)	P-value	Wheezing subjects (n = 452)	Non-wheezing subjects (n = 3373)	P-value
	No. (%)	No (%)		No.	%	
Age (years)						
≤12	218 (74.4)	2611 (74.3)	0.80	337 (75.1)	2503 (74.3)	0.28
13–14	68 (22.9)	825 (23.5)		99 (21.8)	796 (23.6)	
≥14	9 (2.7)	76 (2.2)		15 (3.1)	70 (2.1)	
Gender						
Male	164 (55.6)	1708 (48.6)	0.02	249 (55.1)	1631 (48.4)	0.007
Female	131 (44.4)	1809 (51.4)		203 (44.9)	1742 (51.6)	
Parental education (years)*						
<8	48 (16.4)	564 (16.2)	0.78	68 (15.1)	549 (16.4)	0.57
8–11	195 (66.6)	2383 (63.0)		305 (67.8)	2281 (68.2)	
≥12	50 (17.1)	544 (15.6)		77 (17.1)	517 (15.4)	
Family annual incomes*						
Low	85 (31.1)	1180 (36.3)	0.24	150 (35.5)	1121 (35.9)	0.60
Medium	152 (55.7)	1678 (51.6)		215 (50.8)	1624 (52.1)	
High	36 (13.2)	397 (12.2)		58 (13.7)	374 (12.0)	
Environmental tobacco smoke*						
Yes	138 (47.1)	1655 (47.3)	0.95	224 (49.9)	1575 (46.9)	0.24
No	155 (52.9)	1844 (52.7)		225 (50.1)	1781 (53.1)	
Maternal smoking during pregnancy*						
Yes	18 (6.1)	133 (3.8)	0.05	31 (6.9)	122 (3.6)	0.001
No	277 (93.9)	3384 (96.2)		421 (93.1)	3251 (96.4)	
Cockroaches						
Yes	256 (88.0)	3121 (89.5)	0.43	416 (92.2)	2971 (88.9)	0.03
No	35 (12.0)	368 (10.5)		35 (7.8)	371 (11.1)	
Any home dampness and mould						
Yes	179 (60.7)	1924 (54.7)	0.05	293 (64.8)	1817 (53.9)	0.001
No	116 (39.3)	1593 (45.3)		159 (35.2)	1556 (46.1)	
Duration of breastfeeding (month)*						
0	140 (48.3)	1797 (51.9)	0.55	204 (46.0)	1741 (52.4)	0.04
1–3	120 (41.4)	1327 (38.3)		195 (44.0)	1257 (37.8)	
3–6	18 (6.2)	177 (5.1)		27 (6.1)	169 (5.1)	
≥6	12 (4.1)	163 (4.7)		17 (3.9)	157 (4.7)	
Gestational age (weeks)						
≥37	240 (83.3)	3139 (91.4)	<0.001	56 (12.7)	283 (8.6)	0.005
<37	48 (16.7)	294 (8.6)		385 (87.3)	3010 (91.4)	
Carpet used*						
Yes	26 (8.9)	368 (10.5)	0.39	50 (46.9)	346 (10.3)	0.61
No	266 (91.1)	3135 (89.5)		401 (88.9)	3010 (89.7)	
Pet						
Yes	171 (58.0)	2052 (58.3)	0.90	276 (61.1)	1959 (58.1)	0.23
No	124 (42.0)	1465 (41.7)		176 (38.9)	1414 (41.9)	
Parental atopy						
Yes	135 (45.8)	886 (25.2)	<0.001	195 (43.1)	827 (24.5)	<0.001
No	160 (54.2)	2631 (74.8)		257 (56.9)	2546 (75.5)	

* Number of subjects does not add up to total number because data were missing.

### Air pollution

The distributions of the annual mean air pollutant concentrations in the 14 monitoring stations in the year 2005–2007 are presented in Table 2, and the correlations between different pollutants between communities in Table 3. The correlation between NO_2_ and CO concentrations was high (0.86), which reflects the common source of motor vehicles. The concentrations of PM_2.5_ and SO_2_ were also highly correlated (0.68) indicating common source of stationary fuel combustion, although SO_2_ concentrations were also correlated with both traffic-related pollutants. The concentration of O_3_ was negatively correlated with the mainly traffic-related pollutants, but positively with PM_2.5_ (0.73) and SO_2_ (0.37), and it was only weakly correlated with that of traffic-related and stationary fossil fuel combustion-related air pollutants.

**Table 2:** The mean and distribution of 3-year average air pollutant concentrations between communities, Taiwan 2005–2007.

Pollutant	Mean±SD	Min	Max	Interquartile Range
NO_2_ (ppb)	17.68±0.25	10.06	26.83	8.79
CO (100 ppb)	5.24±0.06	3.04	7.78	1.05
SO_2_ (ppb)	4.33±0.10	2.16	10.09	1.31
PM_2.5_ (µg/m^3^)	33.38±0.50	19.83	51.34	16.84
O_3_ (ppb)	44.64±0.39	30.34	59.12	8.77

Abbreviations: NO_2_, nitrogen dioxide; PM_2.5_, particles with aerodynamic diameter 2.5 µm or less; SO_2_, sulphur dioxide; O_3_, ozone; CO, carbon monoxide.

**Table 3:** Correlations of air pollutants across 14 communities.

	NO_2_	CO	SO_2_	PM_2.5_	O_3_
**NO_2_**	1.00	0.86*	0.55*	0.37	−0.07
**CO**		1.00	0.16	0.09	−0.33
**SO_2_**			1.00	0.68*	0.37
**PM_2.5_**				1.00	0.73*
**O_3_**					1.00

Abbreviations: NO_2_, nitrogen dioxide; PM_10_, particles with aerodynamic diameter 10 µm or less; SO_2_, sulphur dioxide; O_3_, ozone; CO, carbon monoxide.

* Correlation is significant at the 0.05 level.

### Joint effect of air pollution and GSTP1 variants on asthma

The risk of asthma was negatively associated with PM_2.5_ (OR 0.60; 95% CI 0.45, 0.82 per 16.84 µg/m^3^), and O_3_ (OR 0.74; 95% CI 0.60, 0.90 per 8.77 ppb) among Ile-105 homozygotes, but postively associated with PM_2.5_ (OR 1.52; 95% CI 1.01, 2.27 per 16.84 µg/m^3^), and O_3_ (OR 1.19; 95% CI 0.91, 1.57 per 8.77 ppb) among those at least one val105 allele (interaction p value = 0.001 and 0.03, respectively) in single pollutant model (Table 4). In the two-pollutant models, the effects estimates for O_3_ exposure among Ile-105 homozygotes were stable for the three different combinations of pollutants, varying between 0.72 and 0.76. Stable effect estimates (OR = 0.60) was found for PM_2.5_ exposure between single pollutant models and two-pollutant models (Table 5).

**Table 4:** Association between air pollution and asthma and wheezing stratified by GSTP1 genotypes in single pollutant model.

	GSTP1 Ile-Ile		GSTP1 Ile-Val or Val-Val		Interaction p-value
	OR	95% CI	OR	95% CI	
Asthma	(n = 167/2164)		(n = 84/1138)		
NO_2_ (8.79 ppb)	0.88	0.64–1.22	1.30	0.83–2.03	0.24
CO (105 ppb)	1.03	0.89–1.20	1.11	0.90–1.36	0.84
SO_2_ (1.31 ppb)	0.88	0.77–1.01	1.06	0.89–1.26	0.08
PM_2.5_ (16.84 µg/m3)	0.60	0.45–0.82	1.52	1.01–2.27	0.001
O_3_ (8.77 ppb)	0.74	0.60–0.90	1.19	0.91–1.57	0.03
Wheezing	(n = 260/2174)		(n = 137/1141)		
NO_2_ (8.79 ppb)	1.02	0.78–1.32	1.15	0.80–1.65	0.81
CO (105 ppb)	1.08	0.96–1.22	1.07	0.90–1.27	0.67
SO_2_ (1.31 ppb)	0.91	0.81–1.02	1.01	0.87–1.16	0.26
PM_2.5_ (16.84 µg/m3)	0.75	0.59–0.96	1.24	0.89–1.71	0.027
O_3_ (8.77 ppb)	0.82	0.69–0.96	1.09	0.88–1.82	0.049

Two-stage hierarchical analysis adjusting for age, sex, parental education, yearly income, during of breast feeding, gestational age, maternal smoking during pregnancy, environmental tobacco smoke, cockroaches note monthly, carpet, pets, home dampness and mold, parental atopy.

**Table 5:** Association between air pollution and asthma and persistent wheezing stratified by GSTP1 genotypes in two-pollutant models.

**Asthma**	model 1 (O_3_+CO)	model 2 (O_3_+NO_2_)	model 3 (O_3_+SO_2_)	model 4 (PM_10_+CO)	model 5 (PM_10_+NO_2_)
GSTP1: Ile-Ile					
O_3_ (8.77 ppb)	0.72 (0.58–0.89)	0.72 (0.59–0.88)	0.76 (0.62–0.94)		
PM_2.5_ (16.84 µg/m3)				0.60 (0.45–0.82)	0.60 (0.44–0.83)
GSTP1: Ile-Val or Val-Val					
O_3_ (8.77 ppb)	1.30 (0.96–1.77)	1.24 (0.92–1.66)	1.18 (0.89–1.57)		
PM_2.5_ (16.84 µg/m3)				1.52 (1.01–2.27)	1.47 (0.97–2.23)
**Wheezing**					
GSTP1: Ile-Ile					
O_3_ (8.77 ppb)	0.83 (0.69–0.98)	0.81 (0.69–0.96)	0.84 (0.71–0.99)		
PM_2.5_ (16.84 µg/m3)				0.75 (0.59–0.96)	0.74 (0.57–0.95)
GSTP1: Ile-Val or Val-Val					
O_3_ (8.77 ppb)	1.14 (0.90–1.45)	1.10 (0.88–1.38)	1.11 (0.88–1.40)		
PM_2.5_ (16.84 µg/m3)				1.23 (0.89–1.71)	1.21 (0.86–1.70)

Two-stage hierarchical analysis adjusting for age, sex, parental education, yearly income, during of breast feeding, gestational age, maternal smoking during pregnancy, environmental tobacco smoke, cockroaches note monthly, carpet, pets, home dampness and mold, parental atopy.

### Joint effect of air pollution and GSTP1 variants on wheezing

The effect estimates of PM_2.5_ (OR 0.75; 95% CI 0.59, 0.96 per 16.84 µg/m^3^) and O_3_ (OR 0.82; 95% CI 0.69, 0.96 per 8.77 ppb) exposure on the risk of wheezing was substantially reduced among Ile-105 homozygotes compared with among those at least one val105 allele for PM_2.5_ (OR 1.24; 95% CI 0.89, 1.71 per 16.84 µg/m^3^) and O_3_ (OR 1.09; 95% CI 0.88, 1.82 per 8.77 ppb) in single pollutant models (Table 4). The effect estimtes for PM_2.5_ and O_3_ remain constant as we considered different combination of air pollutants in two-pollutant model (Table 5). Our results shows a signifcant joint effect of GSTP1 varant and air pollution particular in PM_2.5_ (interaction p value = 0.027) and O_3_ (interaction p value = 0.049) on wheezing. We did not identify any joint effect between GSTM1 or GSTT1 and air pollutants on asthma/wheezing (Table S2 and Table S3).

## Discussion

There were significantly negative associations between asthma/wheezing and increased the 3-year average concentrations of PM_2.5_ and O_3_ per interquartile changes among Ile-105 homozygotes children. The results provide evidence that children carrying Ile-105 homozygotes are less likely to development of asthma/wheezing as exposure to ambient air pollution, such as PM_2.5_ and O_3_.

### Validity of results

The strength of this study was a large and socio-demographically diverse population of children in Taiwan were included. Unbiased observations of the association between genetic polymorphisms and outcomes were expected. We used routine air-pollution monitoring data as the basis for exposure assessment. These data represented reasonably well exposures both in the school and in the home for two reasons. The schools were chosen to be in the vicinity of the monitoring stations. Almost all the children attended schools within one kilometre of their homes, because the density of middle schools in Taiwan is very high. Finally, the two-stage hierarchical modelling took into account the fact that between communities exposure information and multi-pollutant modes were used. The analytical method is better than previous four studies [4]–[7].

We excluded 286 subjects because of insufficient gene data. This exclusion was unlikely to introduce selection bias, because the characteristic of the excluded individuals did not differ substantially from the included (χ^2^ test; p>0.05), as shown in Table S4. Any known or unknown factors such as air exchange, penetration, deposition as well as emission strengths for indoor pollutants could be responsible for the observed association between personal exposure and municipal level exposure. This was a common limitation in all the previous studies assessing the joint effects of air pollution and genetic variant on the risk of astham or wheezing. A continuous variable of air pollution in this study increases the power to detect interaction, but the number of children with different genotypes who are asthma/wheezing or non-asthma/non-wheezing is still limited. We cannot rule out the possibility of false-negaitve results was obtained.

The outcomes of interstes was based on paretnal report, we cannot rule out the possibility of recall bias. We compared recall of asthma status in a subset of the study population and found that the concordance of parental reports of asthma and medical records' documentation of asthma was good.

Assessment of the independent effects of different pollutants is difficult, because urban air pollution constitutes a complex mixture of several compounds. Although all the measured pollutants have several sources, NO_2_ and CO are predominantly from vehicle emissions whereas the main sources of SO_2_ and PM_2.5_ are stationary fossil combustion processes. In the present study, NO_2_ and CO concentrations were highly correlated indicating the common source of motor vehicle traffic emissions. SO_2_ and PM_2.5_ concentrations were also correlated, their common sources being stationary fossil fuel combustion. In the multi-pollutant modelling, we were able to control for one stationary fossil fuel pollutant at a time as a potential confounder when assessing the effect of one of the traffic-related pollutants and vice versa.

### Synthesis with previous knowledge

The results of the present study, we found negative associations between asthma/wheezing among Ile-105 homozygotes children and exposure to air pollutants particular in PM_2.5_ and O_3_ as continuous scale. Our findings support the present the gene-pollution interaction or effect modification between exposure to air pollutants (PM_2.5_ and O_3_) and GSTP1 variants, especially the isoleucine (Ile)/valine (Val) polymorphism at amino acid position 105 with respect to childhood asthma/wheezing.

Three previous studies from Southern California [5], Cincinnati [6], and Stockholm [4] have elaborated the effect of exposure to outdoor air pollutants on childhood asthma/wheezing appears to be modified by GSTP1. Our present study and Cincinnati study reported a protective effect among children with Ile105 allele of particles (PM_2.5_ or DEP) on the development of wheezing [6]. Stockholm study showed children with at least Val105 allele appear more susceptible to childhood asthma related to traffic-related air pollutant (NOx) [4], but the findings are inconsistent for Southern California study, where Ile105 homozygotes seems to have higher IgE response after O_3_ exposure [5]. Another study in Mexico City reported that asthmatic children with at least Val105 allele are more likely to develop respiratory symptoms as exposure to O_3_ than Ile105 homozygotes [14]. Different gene-pollution interaction in different sittings may occur. It is possible that the pattern of exposure can differ substantially between countries or areas and the genotype frequencies also show large global differences [15]. Whether there are different antioxidant mechanisms of GSTP1 in response to specific air pollutants (CO, CO_2_, NO_2_, SO_2_, DEP, and PM_2.5_) still need to be explored as well as possible differences of effect modification between healthy and asthmatic children.

The biological mechanisms by which the toxic effects of air pollutants (PM_2.5_ and O_3_) seemed to modify the effect of asthma/wheezing are not well understood. After stratification of the GSTP1 genotypes, the protective effect was only in Ile 105 homozygotes. We speculated that GSTP1, might be involved in detoxification of reactive metabolic intermediates and reactive oxygen species (ROS) from air pollution which might be able to protect against oxidative stress [16]–[17].

Exposure of bronchial epithelium to particules may generate reactive oxygen species and increase expression of a marker of oxidative stress, haeme oxygenase 1 [18]. Oxdative stress could be induce by particles promotes inflammation via activation of the transcription factors NF-kB and activator protein-1 and other processes implicated in inflammation, including histone acetylation and the MAPK pathway [19]. In vitro studies have shown that particules increase the production of inflammatory mediators (IL8, GM-CSF and ICAM-1) from human airway epithelia cells [20], which suggest that oxidative stress stimulates the proinflammatory response to particle matter. To our best knowledge, our results first showed that GSTP1 Ile 105 genetically determined antioxidant defense modify the adverse effects of fine “respirable” paticules PM_2.5_ on asthma/wheezing.

Ozone is a strong oxidant and reacts with the epithelial lining fluid to generate free radicals [21]. O_3_ exposure may depletes levles of protective antioxidants (ascorbate and glutathione peroxide) in bronchalveolar lavage fluid [22]. In vitro exposure bronchial epithelia cells to O_3_ indicate increases the production of inflammatiory mediators (IL6, IL8, ICAM-1, granulocyte marcophage-colony stimulating factor (GM-CSF), RANTES and tumour necrosis factor α (TNFα). We speculated that GSTP1 105 Ile, leading to repair detoxification of reactive oxygen species, protected from increased the risk of oxidative stress or inflammation from O_3_.

The possible explanation for our finding was that GST enzyme, largely expressed in human lung cells, act as detoxifying enzymes and serve as a marker of putative oxidative stress [23]. Furthermore, altered antioxidant defenses, lipid peroxidation, and anti-inflammation pathways were important in asthma/wheezing pathogenesis [1]. Overall, our study suggested that genetically predetermined deplete of antioxident enyzme function as a predisposing factor for susceptibililty to air pollutnats (PM_2.5_ and O_3_) and differences in detoxification might contribute to the risk of asthma/wheezing. The differences in direction of effects with GSTP1 genotypes may be due to chance, insufficient power, different populations or ethnic origins, variations of study design and different phenotypes studies. The GSTP1 gene polymorphisms could be a suitable biomarker identifying genetically susceptible children exposed to air pollutants.

### Summary

The present study provides additional evidence that children carrying Ile105 variant allele and exposed to ambient air pollutants, such as PM_2.5_ and O_3_ may be less likely to occurrence of asthma/wheezing. The observed gene-pollution interaction between GSTP1 gene polymorphisms and exposure to PM_2.5_ and O_3_ could help us to understand the aetiology of asthma or wheezing, so allowing earlier prediction and diagnosis of childhood asthma/wheezing, and provide an efficient means of prevention. These results support the roles of genes controlling the antioxidative system and inflammatory response in childhood asthma/wheezing. Diverse detoxification ability of air pollution depended on variations of the GSTP1 polymorphisms. Since air pollution is a complex mixture of compounds and other metabolic genes may be also involved, additional long term prospective study is warranted to explore the relevant role of other genes in determining genetic susceptibility to adverse respiratory outcomes.

## Supporting Information

Table S1 **Primer and MGB probe sequences for** ***GSTT1*** **,** ***GSTM1*** **, and** ***GSTP1*** **genes variants.**
(DOC)

Table S2 **Association between air pollution and asthma and wheezing stratified by GSTM1 genotypes in single pollutant model.**
(DOC)

Table S3 **Association between air pollution and asthma and wheezing stratified by GSTT1 genotypes in single pollutant model.**
(DOC)

Table S4 **Characteristics of included population, excluded population, and total population.**
(DOC)
