# Supplementary material for: Fine Particle, Ozone Exposure, and Asthma/Wheezing: Effect Modification by Glutathione S-transferase P1 Polymorphisms
Source: PLoS One. 2013 Jan 24;8(1):e52715. doi: 10.1371/journal.pone.0052715 (PMC3554722; doi:10.1371/journal.pone.0052715)
Supplement: Table S1 — Primer and MGB probe sequences for GSTT1 , GSTM1 , and GSTP1 genes variants. (DOC) [file pone.0052715.s001.doc]

**Table S1.** Primer and MGB probe sequences for *GSTT1, GSTM1*, and *GSTP1* genes variants.

| Gene | Sequence |
| --- | --- |
| *GSTT1* |  |
| Forward primer | 5’-GTGGTCCCCAAATCAGATGCT-3’ |
| Reverse primer | 5’-GCACCCACGGGCTGT-3’ |
| MGB probe | 5’-(6FAM) CCCTGCCCTCACAACC-3’ |
| *GSTM1* |  |
| Forward primer | 5’- GGAAACAAGGTAAAGGAGGAGTGAT -3’ |
| Reverse primer | 5’-CAAGAATATGTGGGCTGGAACCT -3’ |
| MGB probe | 5’-(6FAM) ACGTGAAGCAAAACAG -3’ |
| *GSTP1* (Ile105Val) |  |
| Forward primer | 5’-CCTGGTGGACATGGTGAATG-3’ |
| Reverse primer | 5’-TGCTCACATAGTTGGTGTAGATGA-3’ |
| MGB probe for Ile allele | 5’-(VIC)CTGCAAATACGTCTCC-3’ |
| MGB probe for Val allele | 5’-(6FAM) TGCAAATACATCTCCCT-3’ |
