# Supplementary material for: Fine Particle, Ozone Exposure, and Asthma/Wheezing: Effect Modification by Glutathione S-transferase P1 Polymorphisms
Source: PLoS One. 2013 Jan 24;8(1):e52715. doi: 10.1371/journal.pone.0052715 (PMC3554722; doi:10.1371/journal.pone.0052715)
Supplement: Table S3 — Association between air pollution and asthma and wheezing stratified by GSTT1 genotypes in single pollutant model. (DOC) [file pone.0052715.s003.doc]

**Table S3.** Association between air pollution and asthma and wheezing stratified by GSTT1 genotypes in single pollutant model.

|  | GSTT1  Null |  | GSTT1  Postive |  | Interaction p-value |
| --- | --- | --- | --- | --- | --- |
|  | OR | CI | OR | CI |  |
| Asthma | (n=155/1661) |  | (n=140/1808) |  |  |
| NO_2_ (8.79 ppb) | 0.94 | 0.66-1.35 | 1.05 | 0.72-1.54 | 0.927 |
| CO (105 ppb) | 1.02 | 0.86-1.21 | 1.07 | 0.89-1.28 | 0.960 |
| SO_2_ (1.31 ppb) | 0.94 | 0.81-1.09 | 0.97 | 0.83-1.14 | 0.661 |
| PM_2.5_ (16.84 µg/m3) | 0.88 | 0.64-1.21 | 0.88 | 0.62-1.25 | 0.895 |
| O_3_ (8.77 ppb) | 0.97 | 0.78-1.20 | 0.87 | 0.60-1.10 | 0.718 |
| Wheezing | (n=235/1585) |  | (n=217/1740) |  |  |
| NO_2_ (8.79 ppb) | 0.82 | 0.60-1.10 | 1.34 | 0.98-1.81 | 0.079 |
| CO (105 ppb) | 0.97 | 0.84-1.12 | 1.17 | 1.02-1.35 | 0.208 |
| SO_2_ (1.31 ppb) | 0.91 | 0.80-1.03 | 0.99 | 0.88-1.12 | 0.270 |
| PM_2.5_ (16.84 µg/m3) | 0.86 | 0.66-1.12 | 0.95 | 0.72-1.25 | 0.455 |
| O_3_ (8.77 ppb) | 0.96 | 0.80-1.15 | 0.90 | 0.75-1.08 | 0.980 |

*Two-stage hierarchical analysis adjusting for age, sex, parental education, yearly income, during of breast feeding, gestational age, maternal smoking during pregnancy, environmental tobacco smoke, cockroaches note monthly, carpet, pets, home dampness and mold, parental atopy.
