# Supplementary material for: Fine Particle, Ozone Exposure, and Asthma/Wheezing: Effect Modification by Glutathione S-transferase P1 Polymorphisms
Source: PLoS One. 2013 Jan 24;8(1):e52715. doi: 10.1371/journal.pone.0052715 (PMC3554722; doi:10.1371/journal.pone.0052715)
Supplement: Table S4 — Characteristics of included population, excluded population, and total population. (DOC) [file pone.0052715.s004.doc]

**Table S4.** Characteristics of included population, excluded population, and total population.

| Characteristic | Included population | Excluded population | Total population | P-value |
| --- | --- | --- | --- | --- |
|  | No. (%) | No (%) | No (%) |  |
| Total | 3816 (100) | 286 (100) | 4102 (100) |  |
| Age (years) |  |  |  |  |
| < 12 | 2992 (78.6) | 224 (78.6) | 3216 (78.6) | 0.55 |
| 13-14 | 804 (21.1) | 59 (20.7) | 863 (21.1) |  |
| > 14 | 12 (0.3) | 2 (0.7) | 14 (0.3) |  |
| Gender |  |  |  |  |
| Male | 1863 (48.8) | 135 (47.2) | 1998 (48.7) | 0.60 |
| Female | 1953 (51.2) | 151 (52.8) | 2104 (51.3) |  |
| Environmental tobacco smoke* |  |  |  |  |
| Yes | 1808 (47.7) | 129 (45.3) | 1937 (47.5) | 0.43 |
| No | 1983 (52.3) | 156 (54.7) | 2139 (52.5) |  |
| Maternal smoking during pregnancy* |  |  |  |  |
| Yes | 156 (4.1) | 10 (3.5) | 166 (4.0) | 0.62 |
| No | 3660 (95.9) | 276 (96.5) | 3936 (96.0) |  |
| Any home dampness and mould |  |  |  |  |
| Yes | 2109 (55.3) | 160 (55.9) | 2269 (55.3) | 0.82 |
| No | 1707 (44.7) | 126 (44.1) | 1833 (44.7) |  |
| Gestational age (weeks) |  |  |  |  |
| >37 | 3378 (90.7) | 249 (88.6) | 3627 (90.6) | 0.24 |
| <37 | 345 (9.3) | 32 (11.4) | 377 (9.4) |  |
| Carpet used* |  |  |  |  |
| Yes | 393 (10.4) | 36 (12.7) | 429 (10.5) | 0.22 |
| No | 3402 (89.6) | 248 (87.3) | 3650 (89.5) |  |
| Pet |  |  |  |  |
| Yes | 2223 (58.3) | 189 (66.1) | 2412 (58.8) | 0.09 |
| No | 1593 (41.7) | 97 (33.9) | 1690 (41.2) |  |

*Number of subjects does not add up to total number because data were missing.
